# Exploring Harms Experienced by Children Aged 7 to 11 Using Ambulance Attendance Data: A 6-Year Comparison with Adolescents Aged 12–17

**DOI:** 10.3390/ijerph15071385

**Published:** 2018-07-02

**Authors:** Debbie Scott, Rose Crossin, Rowan Ogeil, Karen Smith, Dan I. Lubman

**Affiliations:** 1 Eastern Health Clinical School, Monash University, Box Hill, Melbourne, VIC 3128, Australia; rose.crossin@monash.edu (R.C.); rowan.ogeil@monash.edu (R.O.); dan.lubman@monash.edu (D.I.L.); 2 Turning Point, Eastern Health, Richmond, VIC 3121, Australia; 3 Ambulance Victoria, Doncaster, VIC 3108, Australia; karen.smith@ambulance.vic.gov.au; 4 Department of Community Emergency Health and Paramedic Practice, Monash University, Frankston, VIC 3199, Australia; 5 Department of Epidemiology and Preventative Medicine, Monash University, Melbourne, VIC 3004, Australia

**Keywords:** adolescence, self-injury, socio-economic status, suicidal behaviour, substance ingestion, alcohol, prescription drugs, illicit drugs

## Abstract

Many population data sources do not routinely collect data of children under 12, despite research showing that mental health, self-injurious behaviour, and substance ingestion can have severe consequences in this age group. We used 6 years (January 2012 to December 2017) of ambulance attendance data from the Australian state of Victoria to characterise mental health, self-injurious behaviour, and substance ingestion in children aged 7–11. We compared this group to older children aged 12–17. We found that in comparison to those aged 12–17 (*n* = 26,778), a smaller number of children aged 7–11 years (*n* = 1558) were experiencing serious harms, with mental health symptomology the most common harmful outcome. Self-injurious behaviour significantly increased in both age groups throughout the study period. For mental health, self-injurious behaviour and substance ingestion in the 7–11 age group, males were significantly over-represented. These aged 7–11 were more likely to ingest pharmaceuticals, rather than alcohol or illicit substances, and suicidal ideation was the most common self-injurious behaviour in this age group. Our study suggests that data collection needs to occur specifically in the 7–11 age group, and importantly, services and interventions to improve mental health and wellbeing will need to be specifically designed and targeted at this age group.

## 1. Introduction

Over the past two decades, there has been significant investment in youth mental health services worldwide, including headspace in Australia, Youthspace in England, and Headstrong in Ireland. These programs provide youth-targeted services and research to promote mental health and wellbeing, and in the case of headspace, also provide physical health, work and study support, and alcohol and other drug services. Given that adolescence is a high-risk time for the development of mental health and substance use disorders [1,2], with half of all lifetime mental disorders commencing at the age of 14 years, in a cohort of American English-speaking adults with data collected from 2001 to 2003 [3], such initiatives are to be applauded. While the focus of youth services has typically centred on young people aged 12–25 years, with longitudinal studies examining the immediate and long-term outcomes and trajectories of these affected youth [4,5], mental health, substance ingestion and self-injurious behaviour in children aged under 12 are less well-described and understood.

One of the primary reasons for the limited focus on those aged under 12 is a lack of population level data specific to mental health, self-injurious behaviour, and substance ingestion in this age group. Mental health, alcohol and other drug use, and suicidal behaviour have been assessed in the Australian Child and Adolescent Survey of Mental Health and Wellbeing, only conducted between 1998 and 2000 and again in 2013–2014, with no routine data collection. However, students under the age of 11, 12 and 13 were excluded from the mental health section, the suicidal behaviour section and the drug use section of the survey, respectively [6]. The triennial Australian Secondary Schools Alcohol and Drug Survey measures alcohol and other drug use in Australian adolescents, but excludes students under 12 years of age, and does not capture data on the context of substance use (i.e., for recreational or self-harm purposes) [7]. Data are also collected retrospectively and do not capture acute harms. Furthermore, participants may not disclose truthfully, due to fear of stigmatisation, the social desirability of these issues, or consequences if their anonymity is compromised [8]. 

Although these population level surveys have exclusions for those children and young adolescents aged under 11–13, early adolescence does not have clearly defined boundaries, and studies have suggested that harms and health issues typically associated with adolescents are now relevant at a younger age (i.e., the “tweens”) [9,10,11]. More recent evidence shows that for many common mental health disorders, the age of onset can be as low as seven years of age (based on diagnostic interview data from the World Mental Health Surveys) [12]. Suicidal thoughts and behaviour may also emerge from age 11 (from a longitudinal study of American sixth-grade students) [13] or even younger [14,15], though there is a lack of consensus regarding whether suicidal motives can be attributed to children younger than 12 [15,16,17]. In relation to alcohol and other drug use, there are drug types more prone to experimentation by young adolescents, including inhalants [7,18] and analgesics [7]. Both substances have relatively high rates of use in Australian 12-year olds that participated in the Australian Secondary Schools Alcohol and Drugs Survey [7], which would suggest that experimentation has begun at an earlier age, though this is not identified by population level surveys. Collectively, these findings are concerning, given recent research suggesting that harms related to alcohol and drug use, mental health and suicidal behaviour are increasing, and suicide and self-harm is becoming evident at increasingly young ages [19,20,21,22].

One opportunity to overcome existing data issues is to use routinely collected clinical data. Ambulance data is particularly relevant in this context, as not all patients treated by paramedics are transported to hospital, and thus, paramedics may be the first and the only responder to acute harms experienced in the community. Ambulance attendance data have been used previously to examine trends in substance use, mental health and self-harm (e.g., [23,24,25,26]), and represent a unique and alternative means of capturing routine data on children and younger adolescents, who are excluded from population level surveys, while also not being subject to recall bias. The aim of the current study was to describe 6-year trends in the prevalence and characteristics of acute harms related to mental health, self-injurious behaviour and substance ingestion, in children and younger adolescents (aged 7–11 years), who may experience harms [12] but are typically excluded from population level surveys. A further aim was to compare children and younger adolescents with older adolescents (aged 12–17), who are still legally children, but who are relatively well-covered by current data collection, to determine if patterns of behaviour and trends are similar or different between these two age groups.

## 2. Methods

### 2.1. Ambulance Attendance Coding

Data used in this study were from an internationally unique database. Data were provided to Turning Point from Ambulance Victoria (AV), as an output of VACIS^®^ which is an electronic patient care record system. Data extraction has been previously described [24,27,28], but will be briefly outlined below. Initially, the electronic data were parsed to identify relevant alcohol and other drug, and mental health related attendances. This filtered dataset was provided to Turning Point, where case notes were de-identified and transferred into a custom-designed database, and coding was performed by a specialist team of research assistants. Each record was scrutinised, and a systematic and validated coding system used to capture and manually code information held in the clinical notes.

### 2.2. Data for Inclusion

This study utilised data from Victoria only, where attendances occurred between January 2012 and December 2017. For the purpose of this study, children and young adolescents under 18 years were included in the analysis to restrict the data to these who are legally defined as children. Any ambulance attendance for the selected population group that involved ingestion of a pharmaceutical or other substances, or mental health symptomology, or self-injurious behaviour was included. This does not necessarily mean that one of these issues was the primary reason for the ambulance being called, but may have been identified as part of the clinical response of the attending paramedics, and was deemed to have contributed to the reason for the ambulance attendance.

### 2.3. Mental Health Symptomology

Current mental health symptomology is coded rather than mental health diagnosis as paramedics do not screen or assess mental illness diagnoses during an ambulance attendance. The variable ‘Mental health’ is an aggregate of all subtypes of current mental health symptomology. This includes depression, anxiety, psychosis, social or emotional distress, other or unspecified mental health symptomology, and mental health symptomology that can be attributed to physical disease or illness (for example, delusions or hallucinations in delirium).

### 2.4. Self-Injurious Behaviour

“Self-injurious behavior” is an aggregate variable of the subtypes of self-injury and suicidal ideation and behaviour and includes suicide attempt, self-injury, suicidal ideation and self-injury threat, although attendances where a person died by suicide was excluded as it is under-represented in these data as paramedics do not attend all deaths, and where they do, there may not be sufficient data to enable identification of intent. The attribution of intent for self-injurious behaviour is based on reviews of information derived from paramedic assessment of the patient and scene, other evidence found at the scene, patient self-report, and information provided by others at the scene.

### 2.5. Ingestion of Pharmaceuticals or other Substances

Substance ingestion incorporates ingestion of illegal drugs, as well as alcohol, pharmaceuticals, and other substances. The core criterion used in determining the involvement of a pharmaceutical or substance is: “Is it reasonable to attribute the immediate or recent (not merely chronic) over- or inappropriate ingestion of the substance or medication as significantly contributing to the reason for the Ambulance Victoria attendance?”. Substance ingestion was further delineated into alcohol consumption, illicit drug consumption (including inhalant misuse), pharmaceutical misuse, and ingestion of other substances. “Other medication” includes pharmaceuticals (prepared in a pharmaceutical setting) not explicit in the current coding scheme, e.g., prescription medication, over-the-counter medication, vitamins, or herbal medication. These were incorporated into the pharmaceuticals category. “Other substances” includes non-pharmaceutical substances, e.g., methylated spirits or Listerine, which is treated as a separate category.

### 2.6. Statistical Analysis

The project was approved by the Eastern Health Human Research Ethics Committee (E122/0809). Data were de-identified prior to coding, and aggregated for the purpose of this study. Population data were sourced from Estimated Resident Population data from the Australian Bureau of Statistics, with the 2016 age category data used to calculate rates per 100,000 [29]. Socio-economic status (SES) was determined using the 2016 Socio-Economic Indexes for Areas Index of Relative Socio-Economic Disadvantage (SEIFA-IRSD) [30], matched to the patient’s residential postcode. SEIFA-IRSD is a general socio-economic index, based on census data, which summarises a range of information about the economic and social conditions of people and households within an area. A low score indicates relatively greater disadvantage in general, with a high score indicating a relative lack of disadvantage. An area with a low score may have many households with low income, or many people with no qualifications or in low-skill occupations. Relationships between categorical variables were explored using chi-square tests, with *p* values <0.05 considered significant. Trends over time were analysed using linear regression modelling, with time as the independent variable, and monthly ambulance attendance data aggregated into 6-month intervals. Data were not collected from October–December 2014 due to paramedic industrial action, with this missing 3 months of data imputed from the January–September 2014 data, using a rolling 3-month average to estimate trends over time. All data analyses were conducted in STATA (StataCorp. 2013. Stata Statistical Software: Release 13.1., StataCorp LP, College Station, TX, USA).

## 3. Results

Within the study period, there were 1558 ambulance attendances for children aged 7–11, and 26,778 ambulance attendances for those aged 12–17. The younger age group was significantly less likely to be transported to hospital; 59.8% of children aged 7–11 were transported to hospital by paramedics, compared to 77.2% of those aged 12–17 (χ^2^ = 247.55, *p*-value < 0.0001).

### 3.1. What Harms Are Experienced by Younger Children and Adolescents

**Figure 1:**
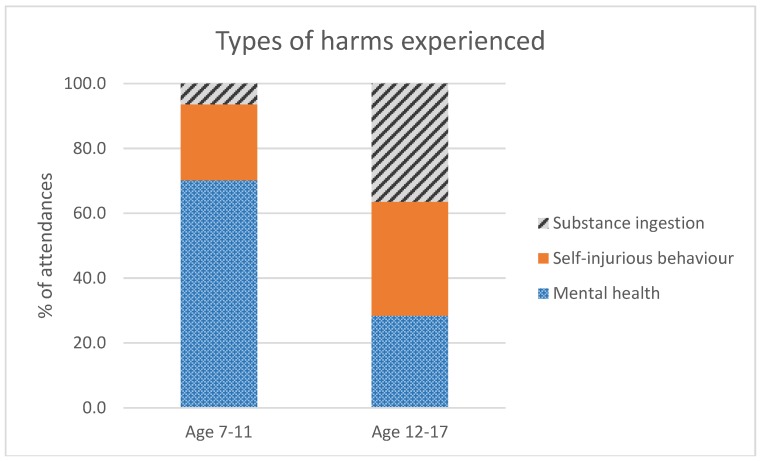
Ambulance attendances as a function of harms experienced. Mental health is the most common outcome experienced by these aged 7–11, followed by self-injurious behaviour and substance ingestion, whereas the inverse of this pattern is seen in those aged 12–17.

Three outcomes (i.e., mental health, self-injurious behaviour, and substance ingestion) were assessed. These outcomes were significantly different between the two age groups (Figure 1). Compared to the 12–17 age group, these aged 7–11 were significantly more likely to have an ambulance attendance related to mental health (χ^2^ = 359.13, *p*-value < 0.0001), but were significantly less likely to have an ambulance attendance for self-injurious behaviour (χ^2^ = 326.93, *p*-value < 0.0001) or substance ingestion (χ^2^ = 1100.00, *p*-value < 0.0001).

### 3.2. Are Rates of Ambulance Attendances Changing over Time?

**Figure 2:**
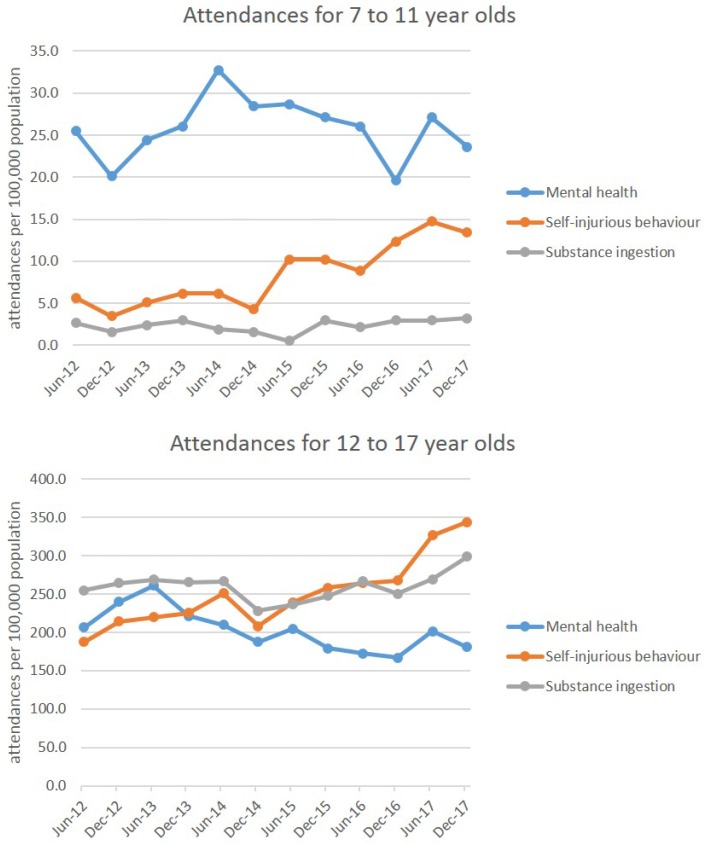
Ambulance attendances between June 2012 and December 2017 for a group aged 7–11 (**top**) and a group aged 12–17 (**bottom**). Ambulance attendances related to mental health and substance ingestion are unchanged over time for these aged 7–11, with a significant increase in attendances related to self-injurious behaviour. For those aged 12–17, ambulance attendances related to self-injurious behaviour also significantly increase over the study period, with no change observed for substance ingestion, and a significant decrease observed for attendances related to mental health.

Rates (per 100,000 population in each age group) for the three outcomes are shown for each age group (Figure 2). For mental health-related ambulance attendances in these aged 7–11, there was no significant trend over time (*R*^2^ = 0.0009, *p*-value = 0.928), compared to a significant decrease over time in attendances for those aged 12–17 (*R*^2^ = 0.52, *p*-value = 0.008). For self-injurious behaviour, there was a similar significant increase in ambulance attendances over the study period for both the 7–11 (*R*^2^ = 0.81, *p*-value < 0.0001) and 12–17 (*R*^2^ = 0.80, *p-*value < 0.0001) age groups. For substance ingestion, there was no significant change over time over the study period for either the 7–11 (*R*^2^ = 0.10, *p*-value = 0.319) or 12–17 (*R*^2^ = 0.06, *p*-value = 0.438) age groups.

### 3.3. Are Gender Patterns Similar between Young and Older Adolescents?

**Figure 3:**
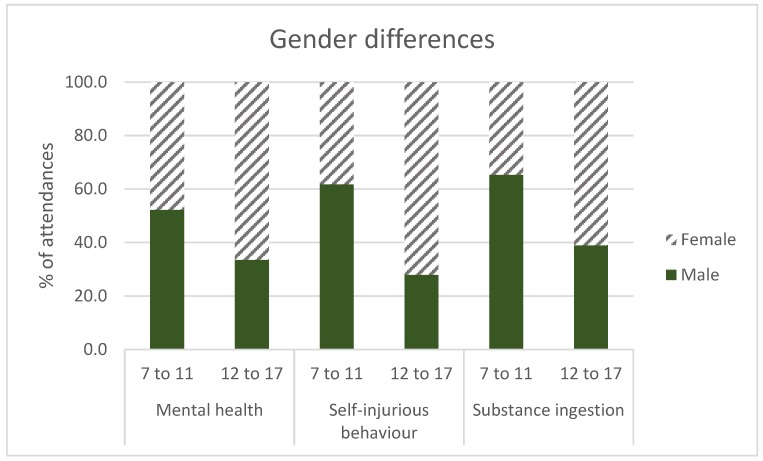
Ambulance attendances in the gender trend between two age groups for mental health, self-injurious behaviour and substance ingestion. Males are significantly over-represented for all three outcomes in the 7–11 age group compared to the 12–17 age group, where females predominate.

There were significant differences of ambulance attendances in the gender trends between the two age groups for all three outcomes (i.e., mental health, self-injurious behaviour and substance ingestion) (Figure 3). For mental health (χ^2^ = 155.47, *p*-value < 0.0001), self-injurious behaviour (χ^2^ = 201.85, *p*-value < 0.0001), and substance ingestion (χ^2^ = 29.54, *p*-value < 0.0001), males were significantly over-represented in the 7–11 age group compared to the 12–17 age group where females predominated.

### 3.4. Is the Influence of Socio-Economic Status the Same between Age Groups?

**Figure 4:**
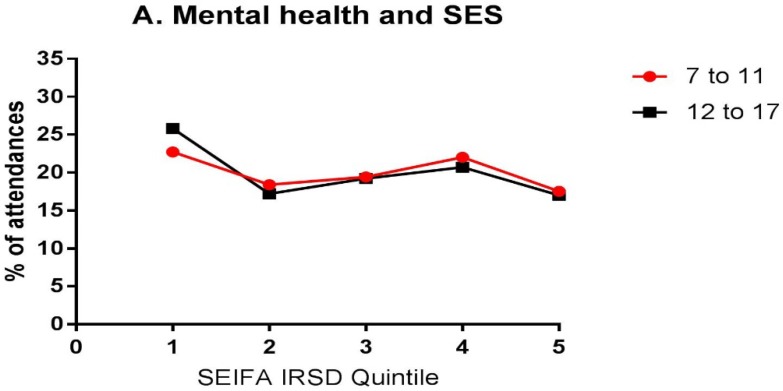

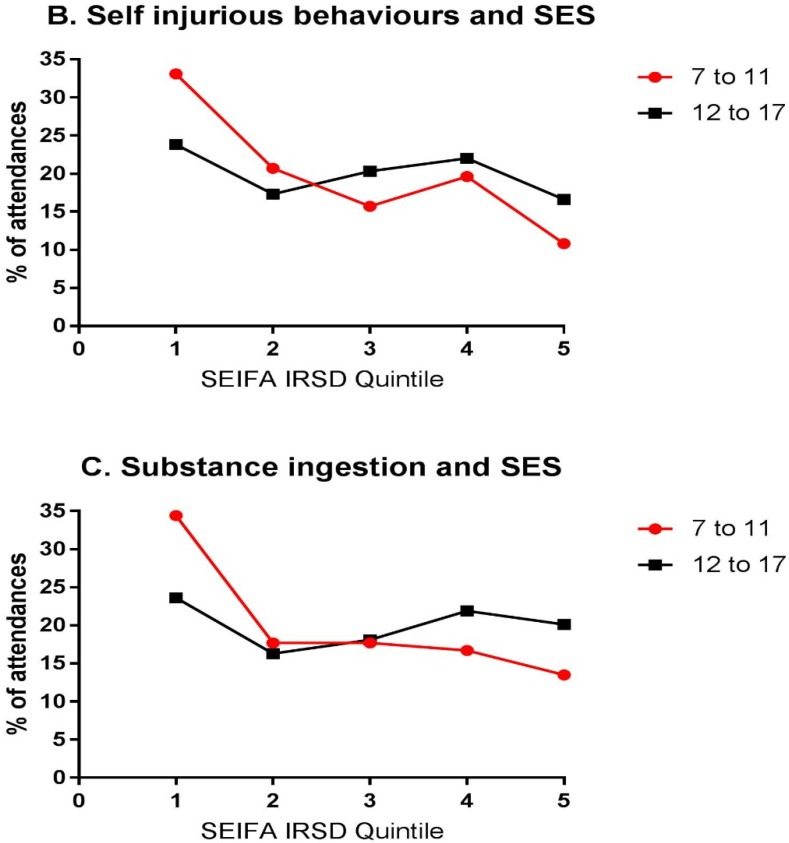
Ambulance attendances as a function of SEIFA-IRSD quintile for mental health (**A**); self-injurious behaviour (**B**); and substance ingestion (**C**). An SES gradient is more evident in the 7–11 age group for self-injurious behaviour, compared to those aged 12–17; however, there are no significant differences for the effect of SES between two age groups on mental health or substance ingestion. SES: Socio-economic status.

For mental health, while there was a slight SES gradient apparent, with ambulance attendances decreasing with the increased SES, there was no significant difference between the age groups (χ^2^ = 5.53, *p*-value = 0.237, Figure 4A). However, for self-injurious behaviour, there was a much stronger SES gradient for the 7–11 age group, with only a slight trend apparent for the 12–17 age group (χ^2^ = 27.01, *p*-value < 0.0001, Figure 4B). For substance ingestion, there was an SES gradient apparent for the 7–11 age group, with no gradient apparent for the 12–17 age group, but this difference did not reach significance (χ^2^ = 8.09, *p*-value = 0.088, Figure 4C).

### 3.5. Do the Two Age Groups Have Similar Patterns in the Types of Substances Ingested?

**Figure 5:**
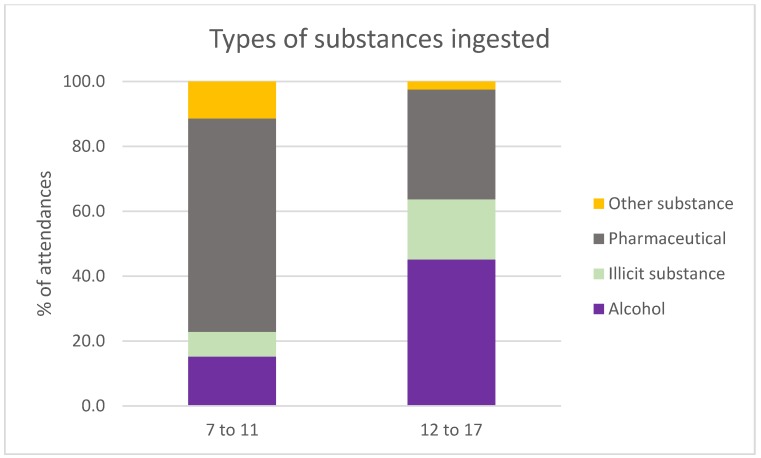
Ambulance attendances as a function of substances ingested. These aged 7 to 11 are more likely to ingest pharmaceuticals and other substances, rather than alcohol or illicit substances, compared with those aged 12–17.

Overall, rates of substance ingestion in these aged 7–11 were low; however, when they experienced acute harms relating to substance ingestion, the rates of substance ingestion were higher than those in the 12–17 age group due to different substances (Figure 5). These aged 7–11 had a higher proportion of ingestion of pharmaceuticals (χ^2^ = 202.22, *p*-value < 0.0001) and other substances (χ^2^ = 3.82, *p*-value = 0.051), whereas those aged 12–17 had a higher proportion of ingestion of alcohol (χ^2^ = 463.93, *p*-value < 0.0001) and illicit substances (χ^2^ = 159.01, *p*-value < 0.0001). 

### 3.6. Do patterns of Self-Injurious Behaviour Differ between Age Groups?

**Figure 6:**
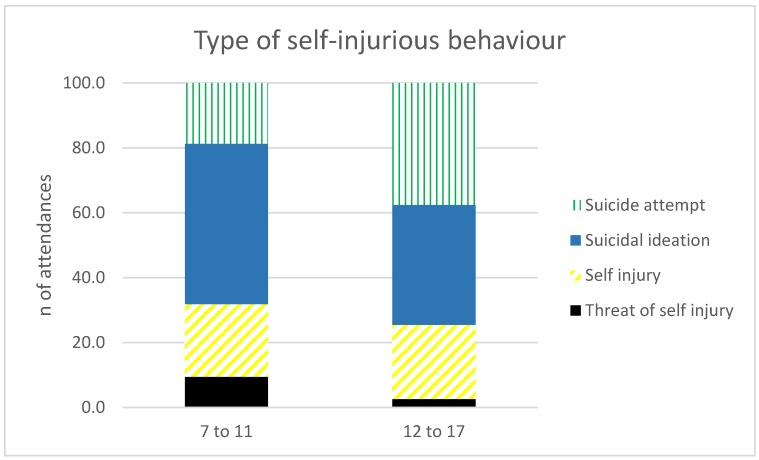
Ambulance attendances as a function of self-injurious behaviour. Threat of self-injury and suicidal ideation are more common in the 7–11 age group, with suicide attempts more common in those aged 12–17.

Self-injurious behaviour increased across both age groups, though the types of self-injurious behaviour differed between groups (Figure 6). These aged 7–11 were more likely to have an ambulance attendance related to a threat of self-injury (χ^2^ = 62.47, *p*-value < 0.0001) or suicidal ideation (χ^2^ = 20.07, *p*-value < 0.0001), with no significant differences for the proportion of those who did self-injure with non-lethal intent (χ^2^ = 0.31, *p*-value = 0.578), and suicide attempts were more prevalent in those aged 12–17 (χ^2^ = 64.12, *p*-value < 0.0001). However, when there were suicide attempts in the younger 7–11 age group, they were significantly more likely to be made using an injurious method (e.g., laceration, hanging, or vehicular impact) (χ^2^ = 82.85, *p*-value < 0.0001), compared to methods involving overdose or self-poisoning that were more prevalent in the 12–17 age group (χ^2^ = 47.27, *p*-value < 0.0001).

## 4. Discussion

This study describes the characteristics of ambulance attendances related to mental health symptomology, or self-injurious behaviour, or substance ingestion, for children aged 7–11, and compared them to a group of older adolescents aged 12–17. Though we recognize that, as a rate per 100,000 population, the incidence of this type of attendance is not high for 7–11 year olds, this represents a cohort of children experiencing significant and acute harms. This age group is not captured by the large Australian population level surveys [6,7]; furthermore, we found that 40% of children who required an ambulance were not transported to hospital, and will therefore not be captured in emergency department data, which provides a further source of data on youth mental health, self-injurious behaviour, and substance ingestion [19,20,21]. It is not clear if the group that is not transported to hospital receives further treatment or support through health services (e.g., a general practitioner, or youth support services) and future research on service provision in this age group would be beneficial. This study highlights that data collection in this age group is necessary, and we recommend that data collection specific to the 7–11 age group be improved.

Importantly, we highlight that in relation to these harms, the 7–11 age group is qualitatively different from the 12–17 age group. These two groups should not be aggregated together, as by absolute numbers, the 7–11 year olds would comprise only 5% of these attendances, and thus, any trends in this age group would be obscured by the older adolescents. Furthermore, it is not possible to impute from the 12–17 age group to the younger children and adolescents. Females predominate in ambulance attendances for all three harms in the 12–17 age group, which is consistent with previous Australian studies looking at emergency department attendances in adolescents aged 12–18 [31] and United States studies looking at emergency department attendances in 11–24 year olds [32]. However, we found that in the 7–11 age group, males were over-represented. We also found that the influence of SES was different between the two age groups, with a stronger effect of decreased SES on increased self-injurious behaviour in these aged 7–11. It has been previously shown that suicidal behaviour has an SES gradient [33], but our study suggests this effect is stronger in younger age groups. This finding is consistent with previous studies that provide evidence for a decreasing influence of family SES as adolescents mature, due to the increased influence of neighbourhood environment and peer groups on health [34]. This stronger influence of SES in the 7–11 age group, however, presents an opportunity in that broader interventions targeted at the level of the family unit may be effective in preventing harms to this age group, e.g., family violence prevention, or alcohol and drug services that may ultimately limit the accessibility of these substances to children.

The type of harms experienced by the 7–11 age group was predominantly mental health related, and rates of mental health related ambulance attendances were unchanged for this age group over the study period, compared to a significant decrease for 12–17 year olds. This is inconsistent with recent studies on emergency department presentations for youth [19,20], but may reflect the definitions used in these studies (i.e., we analysed self-injurious behaviour separately from mental health), or may suggest that adolescents are presenting to emergency departments by means other than an ambulance. Alternatively, it may suggest that the increased focus on youth mental health access and care has been effective, with fewer severe harms (that by their nature may require an ambulance attendance) evident in those aged 12–17, with the lack of targeted interventions and service provision for these aged 7–11 reflected in their unchanged rate of attendances.

Of concern, self-injurious behaviour increased significantly in both age groups. This is particularly worrying for those aged 7–11, because earlier onset of self-injurious behaviour is associated with more severe harms [4], and suicide is a leading cause of death amongst young people worldwide [35]. The 7–11 year olds were more likely to have ambulance attendances for suicidal ideation or threat of self-injury, compared to suicide attempts. This suggests that they are more likely to be engaged in suicidal / self-injurious thoughts, rather than taking action, but this may lead to longer-term issues, given that early onset suicidal ideation is associated with poorer outcomes in adulthood [36]. Future research could focus on longitudinal data collection in this cohort, to determine if morbidity and mortality outcomes are poorer in later adolescence and early adulthood. Males were over-represented in self-injurious behaviour in the 7–11 age group, compared to those aged 12–17, which is consistent with previous studies into suicidal behaviour in childhood [15]. We also found that 7–11 year olds, when they did attempt suicide, did so with more injurious methods, which is consistent with previous studies [15], but may be confounded by the over-representation of males in this age group, as adolescent males are more likely to use injurious and irreversible methods in suicide attempts [37].

The very low rate of attendances for substance ingestion in the 7–11 age group, compared to the 12–17 year olds, is consistent with current conceptualisations of poisoning (as a form of childhood injury), with poisoning mortality data showing a peak in children aged 4 and under, with an increase in mortality from 15 onwards [38]. However, when substance ingestion did occur in the 7–11 age group, the substances were different from the 12–17 year olds. For the 12–17 year olds, the most common types of substances ingested were alcohol and pharmaceuticals, which is consistent with the Australian Secondary Schools Alcohol and Drug Survey [7]. However, these aged 7–11 were more likely to misuse pharmaceutical products and other substances, with a very low proportion of attendances for the ingestion of alcohol or illicit substances. Future research should focus on how these products are sourced, to inform methods for harm reduction or reducing access; furthermore, it would be beneficial for data collection on substance ingestion in this age group to identify the intent behind the ingestion, i.e., experimentation, recreational use, or occurring in the context of self-harm.

It is important to acknowledge a number of limitations with the current study. Findings are based on ambulance attendance data, which are based on the clinical opinion of paramedics supported by information provided by patients and/or witnesses, and this information then goes through secondary coding. Therefore, biases in collection and coding may arise through differences in the way that individual paramedics document the context of an attendance in the electronic patient care record. In addition, ambulance data are collected for operational purposes, and incomplete or inconsistent recording of variables can also occur. Lastly, while this dataset has benefits in that it captures hidden population groups, it will not include data on individuals who self-present to primary care, emergency departments or mental health services. More broadly, however, an ambulance attendance by its nature reflects an individual who is experiencing harm, and as such within the Bronfenbrenner’s Ecological Systems Theory [39], it is focused on the individual. We recognize, however, that for children these harms may be influenced by factors at the microsystem level (e.g., their family groups, or peers), such as housing conditions, parenting, bullying, etc. These ambulance attendance data, while capturing in detail the acute harm(s) experienced by the individual, are not designed to capture this broader context.

## 5. Conclusions

The issue of youth mental health has grown in prominence, with a corresponding increase in tailored interventions and service provision. These efforts have been supported by population level data collection in this age group, which has provided information on the harms experienced and risk factors for these harms. However, children and adolescents under 12 have not had the same level of attention, and data coverage in this age group is limited, which leaves this age group vulnerable to undetected harms. We showed, using ambulance attendance data, that a cohort of children and young adolescents are experiencing serious and increasing harms related to mental health, self-injurious behaviour, and substance ingestion. Importantly, the characteristics of harms in this age group are distinct from older adolescents. This suggests that data collection needs to occur specifically in this age group, without aggregation into older adolescents, and services and interventions to improve mental health and wellbeing will need to be specifically designed and targeted at the 7–11 age group. These examples may include services, such as Kids Helpline that provides counselling services to children for any reason, and services designed to support children within the context of their family (e.g., intensive family support services), but should be guided by future research on harms in children and the underlying causes of those harms. Early onset of these harms is associated with poor outcomes; therefore, it is imperative that it is detected and acted upon in a timely and effective manner when children and young adolescents experience these harms.
